# Importance of Validating Antibodies and Small Compound Inhibitors Using Genetic Knockout Studies—T Cell Receptor-Induced CYLD Phosphorylation by IKKε/TBK1 as a Case Study

**DOI:** 10.3389/fcell.2018.00040

**Published:** 2018-04-10

**Authors:** Marie Lork, Marja Kreike, Jens Staal, Rudi Beyaert

**Affiliations:** Unit of Molecular Signal Transduction in Inflammation, Department of Biomedical Molecular Biology, VIB-UGent Center for Inflammation Research, Ghent University, Ghent, Belgium

**Keywords:** CYLD, IKKε, TBK1, T cell receptor, phosphorylation, kinase inhibitor, antibodies

## Abstract

CYLD is a deubiquitinating enzyme that plays a crucial role in immunity and inflammation as a negative regulator of NF-κB transcription factor and JNK kinase signaling. Defects in either of these pathways contribute to the progression of numerous inflammatory and autoimmune disorders. Therefore, we set out to unravel molecular mechanisms that control CYLD activity in the context of T cell receptor (TCR) signaling. More specifically, we focused on CYLD phosphorylation at Ser418, which can be detected upon immunoblotting of cell extracts with phospho(Ser418)-CYLD specific antibodies. Jurkat T cells stimulated with either anti-CD3/anti-CD28 or PMA/Ionomycin (to mimic TCR signaling) were used as a model system. The role of specific kinases was analyzed using pharmacological as well as genetic approaches. Our initial data indicated that CYLD is directly phosphorylated by the noncanonical IκB kinases (IKKs) IKKε and TANK Binding Kinase 1 (TBK1) at Ser418 upon TCR stimulation. Treatment with MRT67307, a small compound inhibitor for IKKε and TBK1, inhibited TCR-induced CYLD phosphorylation. However, the phospho(Ser418)-CYLD immunoreactive band was still present in CRISPR/Cas9 generated IKKε/TBK1 double knockout cell lines, where it could still be prevented by MRT67307, indicating that the initially observed inhibitory effect of MRT67307 on TCR-induced CYLD phosphorylation is IKKε/TBK1-independent. Most surprisingly, the phospho(Ser418)-CYLD immunoreactive band was still detectable upon immunoblotting of cell extracts obtained from CYLD deficient cells. These data demonstrate the non-specificity of MRT67307 and phospho(Ser418)-CYLD specific antibodies, implying that previously published results based on these tools may also have led to wrong conclusions. We therefore advise to use genetic knockout studies or alternative approaches for a better validation of antibodies and small compound inhibitors. Interestingly, immunoprecipitation with the phospho(Ser418)-CYLD antibody, followed by immunoblotting with anti-CYLD, revealed that CYLD is phosphorylated by IKKε/TBK1 at Ser418 upon T cell stimulation, but that its direct detection with the phospho(Ser418)-CYLD-specific antibody in a western blot is masked by another inducible protein of the same size that is recognized by the same antibody.

## Introduction

Cylindromatosis (CYLD) is a deubiquitinating enzyme that was originally identified as a tumor suppressor in familial cylindromatosis, but has since then also been implicated in other cancer types (Bignell et al., 2000; Massoumi, 2011). The majority of CYLD mutations and truncations found in patients negatively affect its expression or deubiquitinase activity (Massoumi, 2011). Next to its tumor suppressor function CYLD is also a key regulator of immunity and inflammation, which is demonstrated in multiple CYLD genetic mouse models (reviewed in Lork et al., 2017). For example, CYLD knockout mice show defective T cell development and T cells from these mice are hyperresponsive to T cell receptor (TCR)-induced activation and are hyper-proliferative. This hyperresponsive phenotype in CYLD-deficient mice is associated with autoimmune responses and bowel inflammation (Reiley et al., 2006; Zhang et al., 2006). In humans, single nucleotide polymorphisms in the *CYLD* gene have been associated with inflammatory bowel disease (Cleynen et al., 2014). CYLD is a deubiquitinase capable of cleaving K63-linked as well as M1-linked polyubiquitin chains from target proteins (Komander et al., 2009; Ritorto et al., 2014). CYLD negatively regulates nuclear factor kappa-light-chain-enhancer of activated B cells (NF-κB) signaling by removing polyubiquitin chains from specific target proteins including NF-κB essential modifier (NEMO), TNF receptor associated factor (TRAF) 2 and TRAF6, and Transforming growth factor beta-activated kinase 1 (TAK1) (Brummelkamp et al., 2003; Kovalenko et al., 2003; Trompouki et al., 2003; Yoshida et al., 2005; Reiley et al., 2007). In addition, CYLD was shown to negatively affect c-Jun N-terminal kinase (JNK) and p38 mitogen-activated protein kinase (MAPK) signaling pathways, which impacts immune cell function, activation and homeostasis (Yoshida et al., 2005; Zhang et al., 2006; Reiley et al., 2007). Lack of functional CYLD leads to constitutively active downstream NF-κB and MAPK signaling (Reiley et al., 2005; Zhang et al., 2006). Given the importance of CYLD in inflammation and cancer, a better understanding of molecular mechanisms regulating CYLD activity is of considerable interest. CYLD is constitutively expressed in most cell types (Uhlen et al., 2015), suggesting an important role for posttranslational modifications in regulating CYLD activity. Inhibitor of nuclear factor kappa-B kinase (IKK) β- and NEMO-dependent phosphorylation of CYLD on multiple residues within a serine cluster between amino acids 418 and 444 was shown upon stimulation with tumor necrosis factor (TNF), lipopolysaccharide (LPS) and mitogens (Reiley et al., 2005). Other work shows that CYLD can be phosphorylated upon overexpression of the IKK-related kinase IKKε, facilitating IKKε-driven cellular transformation (Hutti et al., 2009).

The serine/threonine kinase IKKε and its homolog TANK binding kinase 1 (TBK1) are referred to as non-canonical IKK kinases as they are closely related to the canonical IKKα and IKKβ, sharing 33% sequence identity within their catalytic kinase domain (Peters et al., 2000; Tojima et al., 2000). IKKε and TBK1 have been intensively studied in the context of type I interferon (IFN) induction in response to viral infection and various pattern recognition receptors, but have also been implicated in the regulation of a number of other processes including autophagy, metabolic regulation and oncogenesis (Shen and Hahn, 2011; Verhelst et al., 2013; Brinkman et al., 2014; Oakes et al., 2017). IKKε/TBK1-mediated type I IFN induction is due to their ability to phosphorylate IFN regulatory factor (IRF) 3 and 7 transcription factors (Fitzgerald et al., 2003; Sharma et al., 2003; Hemmi et al., 2004). Additionally IKKε and TBK1 have been described as NF-κB modulators by phosphorylating IκBα on one of the two critical serines involved in triggering its degradation (Shimada et al., 1999; Bonnard et al., 2000; Peters et al., 2000). Even though TBK1 and IKKε seem to have indistinguishable activities in the activation of IRF3 and IRF7, they do not seem to be fully redundant as they have differential expression patterns and substrate specificities (Fitzgerald et al., 2003; Yu et al., 2012). TBK1 is expressed ubiquitously, while IKKε expression is restricted to particular tissues including the lymphoid tissue, peripheral blood lymphocytes and the pancreas (Shimada et al., 1999; Tojima et al., 2000). TBK1 knockout mice are embryonically lethal and die on embryonic day 14.5 due to massive liver degeneration and apoptosis (Bonnard et al., 2000), while IKKε-deficient mice are viable (Hemmi et al., 2004). TBK1 and IKKε were shown to be activated upon TCR stimulation (Peters et al., 2000; Tojima et al., 2000), but their role in TCR signaling is largely unknown.

We set out to analyze if the noncanonical kinases IKKε and TBK1 regulate TCR-mediated signaling by phosphorylating the deubiquitinase CYLD at Ser418 in Jurkat T cells. Phosphorylation of CYLD at Ser418 was detected upon immunoblotting of cell extracts using phospho(Ser418)-CYLD-specific antibodies. The role of IKKε and TBK1 was assessed using the small compound inhibitor MRT67307 as well as by IKKε/TBK1 double knockout cell lines generated via CRISPR/Cas9. Our data demonstrate that TCR stimulation leads to the phosphorylation of an unknown protein, which is distinct from CYLD but detectable using the phospho(Ser418)-CYLD antibody. Moreover, we show that phosphorylation of this protein can be inhibited by MRT67307 independent of its IKKε and TBK1 inhibitory activity.

## Materials and methods

### Cells, stimulation, transfection

Jurkat T cells were cultured in Roswell Park Memorial Institute (RPMI) supplemented with 10% FCS, 1 mM sodium pyruvate, 2 mM L-glutamine and HEK 293T cells were maintained under standard culturing conditions in Dulbecco's Modified Eagle Medium (DMEM) from Gibco, supplemented with 10% FCS, 1 mM sodium pyruvate, 2 mM L-glutamine. HEK 293T cells were transfected with the indicated plasmids using calcium phosphate and incubated for 24 h at 37°C/5% CO_2_ before further use. IKKε and TBK1 plasmids were a gift from Prof. Alain Chariot (University of Liege, Belgium). The CYLD S418A cDNA was a gift from Jessica Hutti, and was recloned into pCAGGS-E-CYLD (BCCM/GeneCorner plasmid collection; LMBP06613).

IKKε knockout mice were a gift from Prof. Alain Chariot. For isolation of primary cells, spleens were collected from wild-type or IKKε deficient mice. Spleens were injected with 5 ml PBS + 0.5% BSA using a 25 g needle. Spleens as well as washed out cells were passed over a 100 μm cell strainer, which was further rinsed with 5 ml PBS + 0.5% BSA. Cells were spun for 8 min at 1300 rpm at 4°C and supernatant was removed. Red blood cells were lysed in 1.7ml ACK buffer per spleen at room temperature for 4 min. 5 ml PBS + 0.5% BSA per spleen were added and cells were passed over a 40 μm cell strainer and the cell strainer was rinsed with 1 ml PBS + 0.5% BSA. Cells were spun for 8 min at 1,300 rpm at 4°C and the supernatant was removed. The cell pellets were resuspended in 2 ml IMDM supplemented with 5% FCS, β-mercaptoethanol, 20 U/ml penicillin and 20 μg/ml streptomycin per spleen. For CD4^+^ T cells isolation the CD4^+^ T cell Isolation Kit II protocol (Miltenyi Biotec) was used according to the manufacturer's instructions. Cells were stimulated with different stimuli and inhibitors at the indicated concentrations (Table 1).

**Table 1:** Stimuli and inhibitors used.

	**Concentration**	**Cat #**	**Supplier**
**STIMULI**			
PMA	200 ng/ml	P8139	Sigma
Ionomycin	1 μM	CALB407952	Merck millipore
Anti-CD3	10 μg/ml	553057	BD pharmingen
Anti-CD28	10 μg/ml	553294	BD pharmingen
TNF	1,000 U/ml	–	VIB protein service facility
**INHIBITORS**			
MRT67307	1–10 μM	HY-13018	MedChemtronica
TPCA1	5 μM	2559	Tocris biosciences

### Cell lysis and immunoprecipitation

Jurkat T cells or primary CD4^+^ T cells were lysed in Lysis buffer (50 mM Tris HCl, pH 7.4, 150 mM NaCl, 1 mM EDTA, 1% Triton) supplemented with protease and phosphatase inhibitors. Lysates were cleared by centrifugation for 15 min at 14,000 rpm at 4°C. Protein concentration was measured by Bradford protein assay (Bio-Rad). 5x Laemmli buffer (250 mM Tris-HCl pH 8, 10% SDS, 50% glycerol, 0.005% bromophenol blue, 25% β-mercaptoethanol) was added to protein lysates and equal amounts of protein were subjected to immunoblotting. For immunoprecipitation, cell lysates were incubated overnight rotating with specific primary antibodies at 4°C. Then 35 μl of Dynabeads® Protein G (Invitrogen) were added and incubated rotating for 30 min at room temperature (RT). Beads were washed three times with Lysis buffer. Immune complexes were removed from the beads by boiling for 10 min at 90°C in 1x Laemmli buffer and subjected to immunoblotting. For total lysates HEK 293T cells were directly lysed in 1x Laemmli buffer (50 mM Tris-HCl pH 8, 2% SDS, 10% glycerol, 0.001% bromophenol blue, 5% β-mercaptoethanol).

### Immunoblotting

Protein samples were resolved by 8 or 10% SDS-PAGE and proteins were then transferred to 0.45 μm protean nitrocellulose membranes (Whatman) or Immobilon-FL transfer membranes (Millipore). The membranes were blocked in 5% milk powder in TBS/0.2% Tween 20 (TBST) for 1 h at room temperature (RT) and probed with specific primary antibodies overnight at 4°C in 5% milk powder in TBST or 5% BSA in TBST. All antibodies used are listed in Table 2. Membranes were washed three times in TBST and then incubated with HRP-conjugated secondary antibodies (GE healthcare) in 5% milk powder in TBST or 5% BSA in TBST for 1h at RT. Membranes were washed 3 times in TBST and proteins were detected using the Western Lightning ECL detection system (Perkin Elmer Life Sciences) according to the manufacturer's instructions.

**Table 2:** List of antibodies used.

	**MP/BSA**	**Dilution**	**Cat #**	**Supplier**
P-CYLD	BSA	1:1,000	4500	Cell signaling technology
CYLD	MP	1:500	sc-74435	Santa Cruz
P-IKKε	BSA	1:1,000	8766	Cell signaling technology
IKKε	MP	1: 1,000	ab7891	Abcam
P-TBK1	BSA	1: 1,000	5483	Cell signaling technology
TBK1	MP	1: 1,000	3013	Cell signaling technology
P-JNK	BSA	1: 1,000	4668	Cell signaling technology
JNK	MP	1: 1,000	sc-571	Santa Cruz
P-IkBa	BSA	1: 1,000	9246	Cell signaling technology
Actin	MP	1:10,000	MP 6472J	MP biomedicals
Tubulin	MP	1:1,000	T4026	Sigma
E	MP	1:2,500	ab66152	Abcam
Myc	MP	1:3,000		IRC PEP core
Flag	MP	1:1,000	F-3165	Sigma
HA	MP	1:1,000	MMS-101R-B	Babco
anti-mouse IgG HRP	MP	1:3,000	NA931-1ML	Akta
anti-rabbit IgG HRP	MP	1:3,000	NA934V	GEAkta
anti-goat IgG HRP	MP	1:3,000	sc-2020	Santa Cruz

*MP, Milk Powder*.

### *in vitro* kinase assay

250 ng recombinant His-CYLD (#64-0010-050, Ubiquigent) and 35, 70, or 140 ng GST-IKKε (#31177, Active Motif) or GST-TBK1 (#66-0016-050, Ubiquigent) were incubated with 0.6 mM ATP-Mg (#A9187, Sigma) in kinase buffer (20 mM Tris-HCl pH7.5, 10 mM MgCl_2_, 0.1 mM sodiumorthovanadate, 5 mM β-glycerophosphate, 2 mM DTT) for 30 min at 30°C under constant rotation at 550 rpm. Reactions were stopped by adding 5x Laemmli buffer and analyzed by immunoblotting.

### Engineering of knockout cells by CRISPR/Cas9

Knockout cell lines were generated using CRISPR/Cas9 technology as described previously (Ran et al., 2013). Briefly, guide RNAs were designed using the design tool provided by MIT (http://crispr.mit.edu/) and are listed in Table 3. Oligonucleotides (oligos) coding for the guide sequence were extended with CACC in the beginning of the top oligo and CAAA in the end of the bottom oligo. The oligos were phosphorylated and annealed by incubating 1 μl of each oligo (100 μM stock), 1 μl 10x T4 ligation buffer (M1804, Promega) and 1 μl T4 polynucleotide kinase (M4101, Promega) in a total volume of 10 μl in a thermocycler using the following parameters: 37°C for 30 min, 95°C for 5 min, ramp down to 25°C at 5°C/min (=0.1°C/s). The phosphorylated and annealed oligos were diluted 1:200 in ddH_2_O. Oligos were then cloned into pSpCas9(BB)-2A-GFP (48138, Addgene). The reaction mix contained 100 ng pSpCas9(BB)-2A-GFP, 2 μl oligo, 2 μl 10x Tango buffer (Fermentas), 0.5 mM DTT, 0.5 mM ATP, 1 μl FastDigest BbsI (FD1014, Fermentas) and 0.5 μl T4 ligase (M1804, Promega) in a total volume of 20 μl. The reaction was incubated for 6 cycles: 5 min at 37°C followed by 5 min at 21°C. Plasmids were transformed into DH5α competent *E. coli*. Plasmid DNA was extracted using the PureLink® Quick Plasmid Miniprep Kit (K210010, Invitrogen) according to the manufacturer's instructions and the sequences were verified by Sequencing from the U6 promoter using the U6-Fwd primer (GAGGGCCTATTTCCCATGATTCC) to check that the 20 nt guide sequence is inserted between the U6 promoter and the remainder of the sgRNA scaffold. The plasmids (2 μg) were transfected into 2 × 10^6^ Jurkat T cells using Amaxa cell line nucleofector kit V (VCA-1003, Lonza) using program X5 according to the manufacturer's instructions. Three days after transfection GFP positive single cells were sorted into 96 well plates using a BD FACS ARIA III (BD Bioscience). Single cell clones were expanded and knockout was confirmed on protein level by immunoblotting and on genomic level by amplification of the region of interest followed by sequencing. For this, genomic DNA was extracted using QuickExtract DNA Extraction Solution (QE09050, Epicenter) according to the manufacturer's instructions and the region of interest was amplified using the primers indicated in Table 3 with the KAPA HiFi HotStart Readymix (KK2602, kapa biosystems) according to the manufacturer's instructions. PCR products were purified using CleanPCR beads (CPCR-0050, Cleanna) according to the manufacturer's instructions and sequenced using the primers indicated in Table 3. The sequences were analyzed using the Tracking of Indels by DEcomposition (TIDE) software (Brinkman et al., 2014).

**Table 3:** Oligos and primers used for CRISPR/Cas9 mediated geneome editing.

	**Primer**	**Sequence**
CYLD	Guide top oligo	CACCAGAGTGGAATCTGTTCTCGG
	Guide bottom oligo	AAACCCGAGAACAGATTCCACTCT
	Forward Primer	ACAAAGACTATCACGTATACGATACCCG
	Reverse Primer	TTAACTTCAGCCAATGAGCCCA
	Sequencing Primer	AGGAGCGAGAACACTGTT
IKKε	Guide top oligo	CACCGAGAAGTTCGTCTCGGTCTA
	Guide bottom oligo	AAACTAGACCGAGACGAACTTCTC
	Forward Primer	CACCCATCTTGGTTTCCTAGAGAA
	Reverse Primer	AGCCTCACCTTTTCCATCTTAGAGAAC
	Sequencing Primer	ATGGCTCTGTCAGCCCAT
TBK1	Guide top oligo	CACCCATAAGCTTCCTTCGTCCAG
	Guide bottom oligo	AAACCTGGACGAAGGAAGCTTATG
	Forward Primer	GATGATTTTGCTTTTGATACTATTATGGC
	Reverse Primer	GGTTGTACAAACCCTAATTTTCAACAGT
	Sequencing Primer	TATCCATTTCTGAATTCC

## Results

### IKKε and TBK1 are activated upon T cell activation and can directly phosphorylate CYLD at Ser418

**Figure 1:**
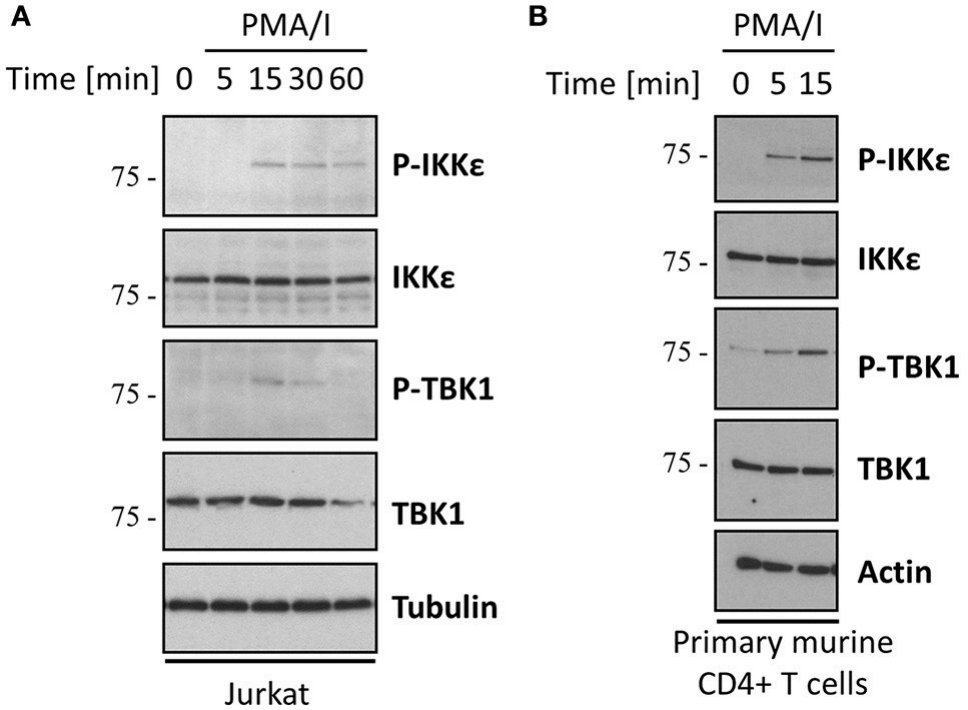
The noncanonical kinases IKKε and TBK1 are activated upon TCR stimulation. Jurkat T cells **(A)** or primary mouse CD4^+^ T cells **(B)** were stimulated with 200 ng/ml PMA and 1 μM Ionomycin for the indicated times. Protein levels were determined by immunoblotting. Activation of IKKε and TBK1 was determined using an antibody specifically recognizing their phosphorylation at Ser172. Results shown are representative for three **(A)** and two **(B)** independent experiments.

**Figure 2:**
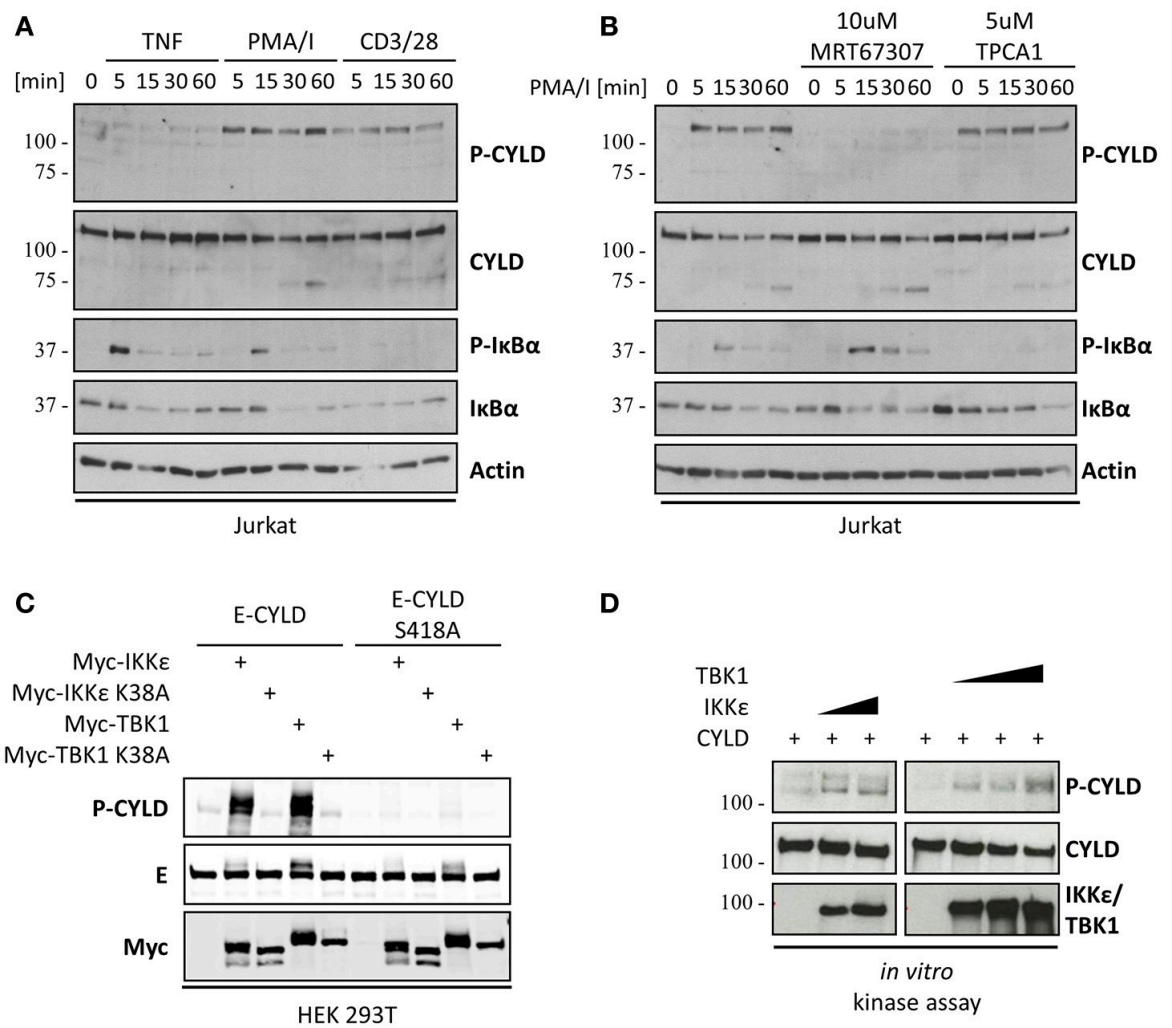
The deubiquitinase CYLD is phosphorylated at Ser418 by IKKε and TBK1. **(A)** CYLD phosphorylation at Ser418 is observed upon TCR- but not TNFR stimulation. Jurkat T cells were stimulated with 200 ng/ml PMA and 1 μM Ionomycin or 20 μg/ml anti-CD3 and anti-CD28 or 1,000 U/ml TNF for the indicated times. **(B)** CYLD phosphorylation is abolished by IKKε/TBK1, but not IKKβ inhibition Jurkat T cells were stimulated with 200 ng/ml PMA for the indicated times. Cells were pre-incubated in the presence or absence of 10 μM IKKε/TBK1 inhibitor MRT67307 or 5 μM IKKα/β inhibitor TPCA1. **(C)** IKKε/TBK1 co-expression induced CYLD phosphorylation. HEK 293T cells were transfected with the indicated expression constructs and harvested 24 h post transfection. **(D)** CYLD is phosphorylated by IKKε and TBK1 *in vitro*. 250 ng recombinant His-CYLD was incubated with 35 or 70 ng GST-IKKε IKKε or 35, 70, or 140 ng TBK1 in an *in vitro* kinase assay **(A–D)** Protein levels were determined by immunoblotting. Results shown are representative at least three independent experiments.

IKKε and its homolog TBK1 were previously shown to be activated upon crosslinking of CD3 and the co-stimulatory receptor CD28 (Yu et al., 2015). In agreement, we found that treatment of the Jurkat T cell line with a combination of phorbol myristate acetate (PMA) and Ionomycin (I), which mimics TCR signaling by activating PKC and calcium influx, results in the activation of IKKε and TBK1 as assessed by immunoblotting with an antibody that specifically recognizes Ser172 phosphorylation in the IKKε/TBK1 activation loop (Kishore et al., 2002). Similar results were obtained in primary murine CD4^+^ T cells (Figure 1). We next investigated if CYLD can be phosphorylated by IKKε or TBK1 in the context of TCR signaling. We focused on CYLD phosphorylation at Ser418, which can be easily assessed using a phospho(Ser418)-CYLD-specific antibody. Stimulation of Jurkat T cells with anti-CD3/CD28 or PMA/I led to the rapid phosphorylation of CYLD at Ser418 (Figure 2A). The faster migrating band in the case of immunoblotting for total CYLD corresponds to the previously reported CYLD cleavage product generated by MALT1 (Staal et al., 2011). Although TNF was previously shown to trigger CYLD phosphorylation in Jurkat, HeLa, or HEK 293 cells, leading to the appearance of a slower migrating form of CYLD (Reiley et al., 2005), stimulation of our Jurkat T cells with TNF did not lead to a band shift or detection of a band with the phospho(Ser418)-specific antibody, suggesting either no phosphorylation or phosphorylation at other sites. It should be noted however, that a phospho-CYLD specific signal was detected in several other Jurkat cell clones tested (Figure S1), indicating clonal differences. The TCR- and PMA/I-induced Ser418 phosphorylation of CYLD could be completely prevented by dual inhibition of IKKε and TBK1, using MRT67307, while the IKKβ inhibitor TPCA1 had no effect (Figure 2B). CYLD Ser418 phosphorylation was also observed upon co-expression with IKKε or TBK1, but not upon co-expression of their catalytically inactive counterparts (K38A) in HEK 293T cells (Figure 2C). When the Ser418 residue in CYLD was mutated to alanine, we could no longer detect its IKKε/TBK1-mediated phosphorylation using the phospho(Ser418)-CYLD-specific antibody, confirming phosphorylation at Ser418. However, we still detected a band shift using an antibody against the epitope-tag fused to CYLD, indicating the presence of additional IKKε/TBK1-mediated phosphorylation sites in CYLD upon overexpression of the kinases. Further, in an *in vitro* kinase assay we could show that recombinant IKKε and TBK1 can directly phosphorylate CYLD at Ser418 (Figure 2D). Together these data suggest that T cell activation is associated with the IKKε/TBK1 mediated phosphorylation of CYLD at Ser418.

### The IKKε/TBK1 inhibitor MRT67307 inhibits CYLD phosphorylation independent of IKKε and TBK1

**Figure 3:**
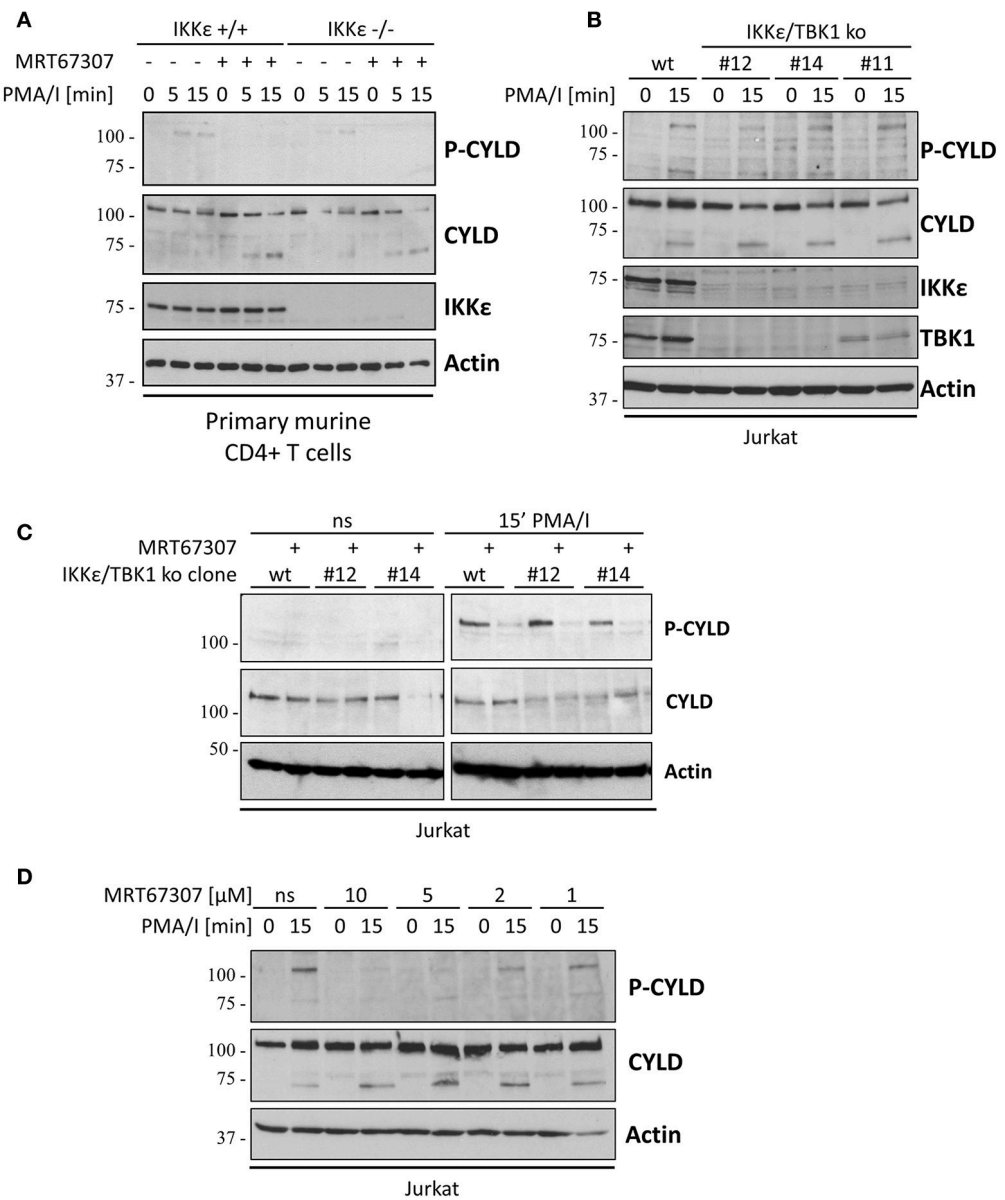
The TCR-induced phospho(Ser418)-specific band occurs independent of IKKε and TBK1 **(A)** Primary mouse CD4^+^ T cells from wild-type (wt) or IKKε knockout (ko) mice were pre-incubated for 30 min in the presence of absence of 10 μM IKKε/TBK1 inhibitor MRT67307 and subsequently stimulated with 200 ng/ml PMA and 1μM Ionomycin for the indicated times. Specific proteins were determined by immunoblotting with the indicated antibodies. **(B)** Wild-type (wt) or IKKε/TBK1 double deficient Jurkat T cell clones were stimulated with 200 ng/ml PMA and 1 μM Ionomycin for 15 min. Specific proteins were determined by immunoblotting with the indicated antibodies. **(C)** Wt or IKKε/TBK1 double deficient Jurkat T cell clones were pre-incubated for 30 min in the presence or absence of 10 μM IKKε/TBK1 inhibitor MRT67307 and then stimulated with 200 ng/ml PMA and 1 μM Ionomycin for 15 min. Specific proteins were determined by immunoblotting with the indicated antibodies. **(D)** Wt Jurkat cells were pre-incubated for 30 min in the presence of 1, 2, 5, or 10 μM IKKε/TBK1 inhibitor MRT67307 and then stimulated with 200 ng/ml PMA and 1 μM Ionomycin for 15 min. Specific proteins were determined by immunoblotting with the indicated antibodies. The data are representative of at least two independent experiments.

As MRT67307 blocks both IKKε and TBK1, we wanted to determine which of the two kinases is responsible for CYLD phosphorylation. Therefore, we first tested PMA/I-induced CYLD phosphorylation in primary CD4^+^ T cells from IKKε deficient mice. As a control, wild-type and IKKε deficient cells were stimulated in the presence of MRT67307. MRT67307 significantly decreased CYLD phosphorylation in wild-type cells (Figure 3A). However, there was no difference in CYLD phosphorylation between wild-type and IKKε knockout cells. Moreover, CYLD phosphorylation was still inhibited by MRT67307 in IKKε deficient cells. Together, these results suggest that TCR-induced CYLD phosphorylation is independent of IKKε. Alternatively, the effect of IKKε deficiency may be masked by the redundant function of TBK1. To further investigate a possible redundancy between IKKε and TBK1, we generated IKKε and TBK1 double deficient Jurkat T cells using CRISPR/Cas9 technology. Several independent clones were derived from single cells. Knockout of IKKε and TBK1 was confirmed by immunoblotting as well as by genomic sequencing of the region of interest. Unexpectedly, a phospho(Ser418)-CYLD signal was still detectable upon PMA/I stimulation in the IKKε/TBK1 double deficient Jurkat cells, indicating that a kinase different from IKKε and TBK1 is responsible for CYLD phosphorylation (Figure 3B). Furthermore, this signal could be inhibited by pre-treatment with MRT67307, suggesting that this inhibitor acts independently of IKKε/TBK1 (Figure 3C). As it was shown previously that MRT67307 can inhibit IKKε and TBK1 at concentrations as low as 1–2 μM (Clark et al., 2011a), the concentration of 10 μM used may have led to off-target effects. Therefore we tested if the phospho(Ser418)-CYLD signal could also be inhibited when using MRT67307 at lower concentrations (Figure 3D). Here we could show that 5 μM MRT67307 completely prevented CYLD phosphorylation, while 1 or 2 μM reduced the phospho(Ser418)-CYLD signal only partially (Figure 3D). Taken together, these results indicate that MRT67307 inhibits the phospho(Ser418)-CYLD signal independent of its IKKε and TBK1 inhibitory properties.

### The phospho(Ser418)-CYLD-specific antibody detects a protein distinct from CYLD

**Figure 4:**
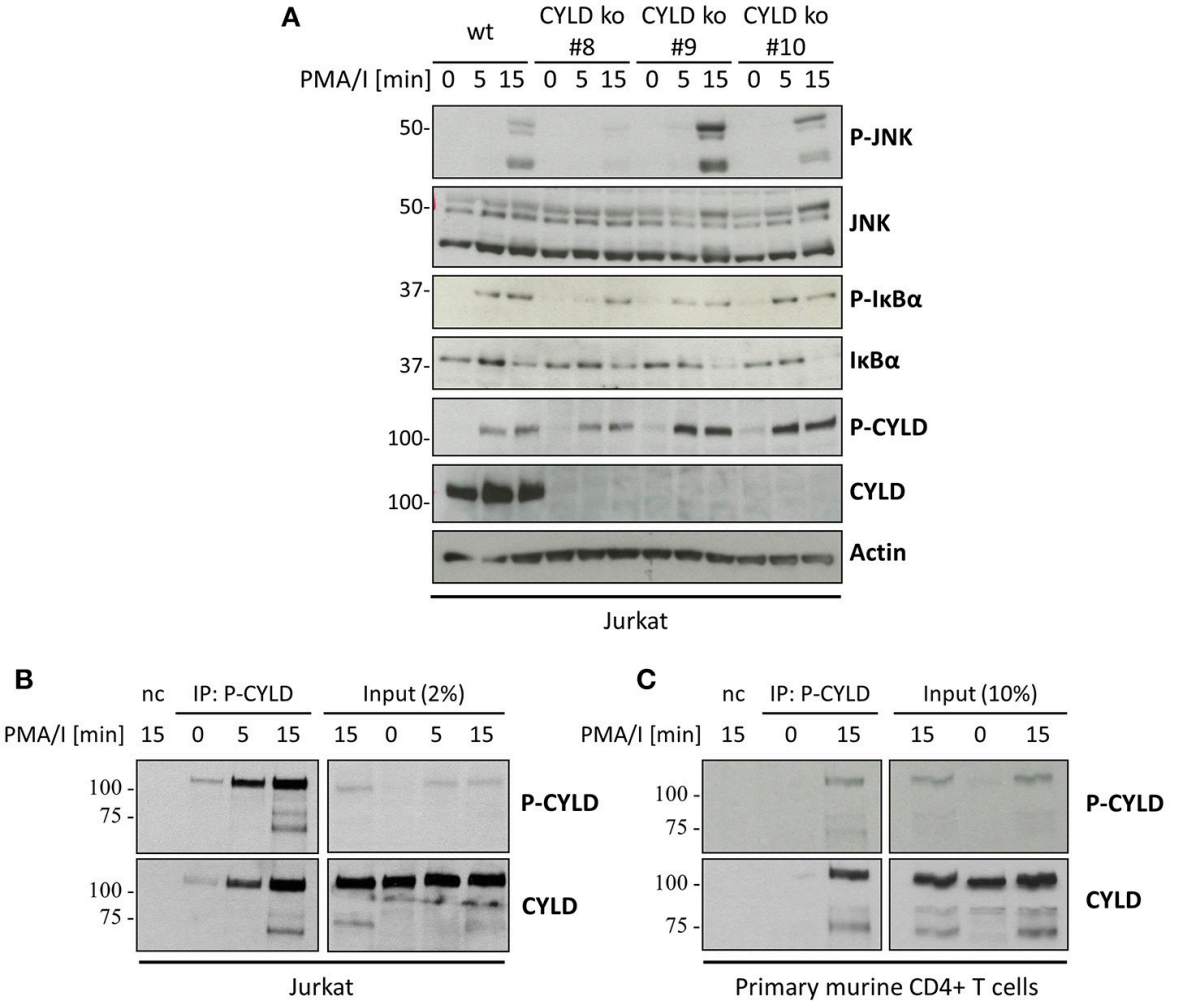
The phospho(Ser418)-CYLD specific antibody still detects a protein of the same size as CYLD in CYLD-deficient cells. **(A)** Wild-type (wt) or three different CYLD knockout (ko) Jurkat T cell clones were stimulated with 200 ng/ml PMA and 1 μM Ionomycin for the indicated times. **(B)** Jurkat cells or **(C)** primary murine CD4^+^ T cells were stimulated with 200 ng/ml PMA and 1 μM Ionomycin (PMA/I) for the indicated times. Cell extracts were subjected to immunoprecipitation using the phospho(Ser418)-CYLD-specific antibody. Beads without antibody were used as negative control (nc). **(A–C)** Specific proteins were determined by immunoblotting with the indicated antibodies.

To further elucidate the importance of CYLD phosphorylation at Ser418 for proximal TCR signal transduction, we generated CYLD knockout cells by CRISPR/Cas9 to subsequently reconstitute with CYLD(S418A) or CYLD(S418E) mutants that prevent or mimic CYLD phosphorylation at Ser418, respectively. Absence of CYLD was confirmed by immunoblotting as well as genomic sequencing. Three different CYLD-deficient Jurkat T cell clones were characterized for signaling in response to PMA/I. While all clones show similar inducible IκBα phosphorylation, they differ strongly in JNK activation (Figure 4A). JNK phosphorylation in clone #10 is comparable to wild-type Jurkat T cells, while clone #8 shows decreased and clone #9 increased JNK phosphorylation. This clonal variation between the different CYLD deficient Jurkat T cell clones illustrates that one should be highly cautious when using specific knockout cell clones generated by CRISPR/Cas9 and that the effect of CYLD reconstitution should be tested in order to make valid conclusions on the impact of CYLD deficiency on JNK activation. The latter was beyond the scope of the current study. Most surprisingly, however, western blotting and detection with the phospho(Ser418)-CYLD-specific antibody still revealed the same PMA/I-inducible phospho(Ser418)-CYLD signal in all CYLD-deficient cell clones (Figure 4A). These data indicate that the phospho(Ser418)-CYLD-specific antibody detects a protein distinct from CYLD, but with the same molecular weight of approximately 110 kDa. The impact of this finding on our current data and previously published results of others using the same antibody will be discussed below. From Figure 2C it is obvious that this antibody specifically recognizes CYLD phosphorylation at Ser418 upon overexpression. At this point, it is not possible to exclude that the unspecific 110 kDa band masks a specific phospho-CYLD band in the Jurkat cell line. We therefore performed an immunoprecipitation using the phospho(Ser418)-CYLD-specific antibody, followed by western blotting with a CYLD antibody (Figures 4B,C). Indeed, CYLD could be readily detected upon immunoprecipitation with the phospho(Ser418)-CYLD-specific antibody from PMA/I-stimulated Jurkat T cells and primary murine CD4^+^ T cells, showing that CYLD is indeed phosphorylated at Ser418 upon T cell stimulation but that its direct detection with the phospho(Ser418)-CYLD-specific antibody in a western blot is masked by another inducible protein of the same size that is recognized by the same antibody.

**Figure 5:**
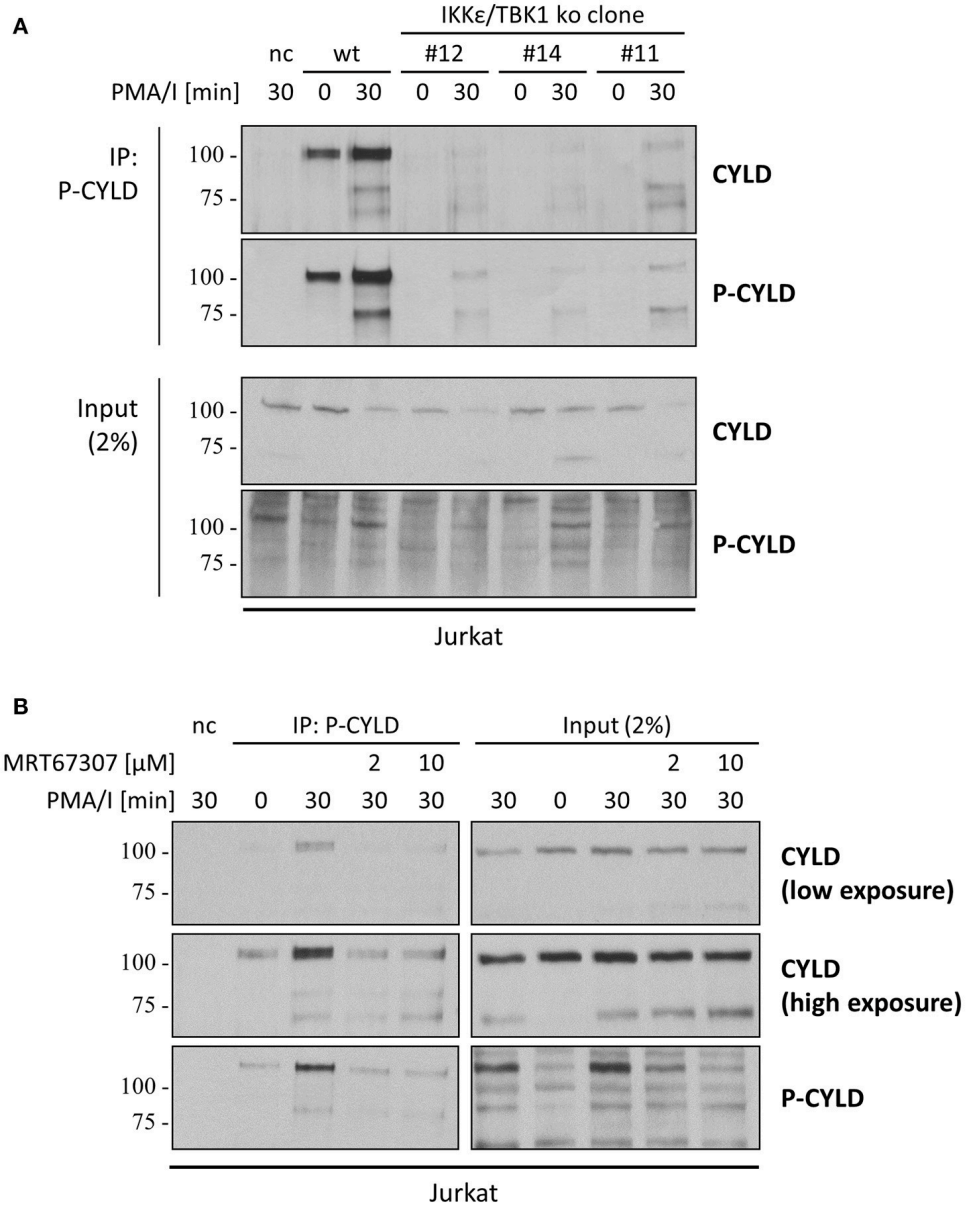
CYLD is phosphorylated in an IKKε/TBK1 dependent manner, which can be detected by immunoprecipitation of CYLD using the phospho(Ser418)-CYLD-specific antibody. **(A)** Wild-type (wt) or IKKε/TBK1 double knockout (ko) Jurkat T cell clones were stimulated with 200 ng/ml PMA and 1 μM Ionomycin (PMA/I) for 30 min. **(B)** Wt Jurkat T cells were pre-incubated for 30 min in the presence of 2 or 10 μM IKKε/TBK1 inhibitor MRT67307 and then stimulated with 200 ng/ml PMA and 1μM Ionomycin for 30 min. **(A,B)** Cell extracts were subjected to immunoprecipitation using the phospho(Ser418)-CYLD-specific antibody. Beads without antibody were used as negative control (nc). Specific proteins were determined by immunoblotting with the indicated antibodies.

To determine if the specific phosphorylation of CYLD is dependent on IKKε and TBK1 as hypothesized earlier, we performed an immunoprecipitation with the phospho(Ser418)-CYLD-specific antibody in IKKε/TBK1 deficient cells (Figure 5A). While we could detect immunoprecipitated CYLD in PMA/I stimulated wild-type Jurkat T cells, we did not detect any CYLD upon immunoprecipitation with the phospho(Ser418)-CYLD-specific antibody in the IKKε/TBK1 knock-out cells, indicating that CYLD phosphorylation is indeed dependent on these two kinases. Interestingly, we could detect a band with the phospho(Ser418)-CYLD-specific antibody in the total lysate control of the IKKε/TBK1 knock-out clones (Figures 3B,C, 5A), indicating that this antibody specifically detects phosphorylated CYLD under native conditions, while it additionally detects an unknown protein of the same size in denaturing conditions. We further confirmed the importance of IKKε and TBK1 for CYLD phosphorylation by treatment of cells with MRT67307, followed by immunoprecipitation of cell lysates with the phospho(Ser418)-CYLD-specific antibody (Figure 5B). Here we show that specific phosphorylation of CYLD upon immunoprecipitation is already completely inhibited at 2 μM MRT67307 (Figure 5B, left panel), while 10 μM MRT67307 is necessary to completely prevent the phosphorylation band in the total lysate (Figure 5B, right panel). Taken together these data show that CYLD is phosphorylated upon TCR stimulation and that this phosphorylation is dependent on IKKε and TBK1. However, the CYLD phosphorylation band in total cell lysates is masked by the phosphorylation of another protein of the same size which is also detected by the phospho(Ser418)-CYLD-specific antibody. Specific CYLD phosphorylation should thus be studied by immunoprecipitation with this antibody followed by CYLD detection via immunoblotting.

## Discussion

In the present study we show that co-expression of CYLD and IKKε/TBK1 in HEK293T cells, as well as incubation with recombinant IKKε/TBK1, results in CYLD phosphorylation that can be detected with a phospho(Ser418)-CYLD-specific antibody upon immunoblotting. A similar signal, sensitive to MRT67307, could be detected in TCR-stimulated Jurkat cells. CYLD is known to be phosphorylated on a serine cluster (amino acids 418-444) in an IKKβ/NEMO-dependent manner in response to TNF, LPS and PMA/I, resulting in a band shift upon immunoblotting (Reiley et al., 2005). It must be noted that we did not observe such a band shift of CYLD upon TCR crosslinking or PMA/I stimulation of Jurkat T cells, while we did detect a clear signal using the phospho(Ser418)-CYLD antibody. At the functional level, CYLD phosphorylation was previously shown to prevent CYLD-mediated TRAF2 deubiquitination and to promote TNF-induced gene expression (Reiley et al., 2005). Similarly, CYLD Ser418 phosphorylation upon co-expression of IKKε was found to decrease its DUB activity (Hutti et al., 2009). IKKε-dependent CYLD Ser418 phosphorylation and inactivation of its DUB activity has also been reported in response to stimulation of the C-type lectin receptor DC-SIGN in dendritic cells (Gringhuis et al., 2014). Remarkably, there is also a contradictory report showing that CYLD Ser418 phosphorylation increases its *in vitro* DUB activity toward K63-linked polyubiquitin (Thein et al., 2014). Surprisingly, using CYLD deficient cells we found that the phospho(Ser418)-CYLD antibody cross-reacts with another unknown protein of the same size as CYLD, and whose phosphorylation is also inhibited by MRT67307. These findings not only forced us to reevaluate our findings on TCR-induced CYLD phosphorylation, they also imply that one should be very cautious with the interpretation of several published findings using the phospho(Ser418)-CYLD antibody (Table 4). However, by immunoprecipitation with the phospho(Ser418)-CYLD-specific antibody followed by CYLD detection via immunoblotting and detection with anti-CYLD, we were able to detect specific CYLD phosphorylation, and could show that this was indeed dependent on IKKε and TBK1, confirming our initial hypothesis.

**Table 4:** Publications related to CYLD phosphorylation on Ser418.

**References**	**Kinase**	**Context**	**Effect**
Beli et al., 2012	nd	TNF, ionizing radiation or etoposide treatment of U2OS cells	nd
Gringhuis et al., 2014	IKKε	DC-SIGN stimulation of dendritic cells	Reduced DUB activity; reduced Bcl3 deubiquitination and nuclear translocation
Thein et al., 2014	IKKα/β	Postsynaptic density	Increased DUB activity
Zhu et al., 2014	IKKε/TBK1	Co-expression with TBK1 or IKKε in HEK 293 cells, abrogated by MRT67307 treatment	nd

*nd, not determined*.

Antibodies are among the most frequently used research tools in basic science and are used in a wide range of applications. Many antibodies are however not properly validated. One of the pitfalls is that detection of a single band of the correct molecular weight on a western blot is often used to demonstrate specificity. However, our results with a commonly used phospho(Ser418)-CYLD antibody demonstrate this is far from true and that there is a clear need for universal standards in antibody validation. The International Working Group for Antibody Validation (IWGAV) proposed a model with standard guidelines for antibody validation based on five conceptual pillars or validation strategies (Uhlen et al., 2015). First, the antibody should be tested on cells in which expression of a protein of interest is reduced or eliminated by RNA interference or knockout using techniques such as CRISPR/Cas9. Second, orthogonal antibody-independent methods such as mass spectrometry or qPCR can validate expression. Third, protein expression can be compared using two distinct antibodies that recognize non-overlapping epitopes. Fourth, the expression of a protein that contains an epitope-tag (e.g., Flag, His) can be analyzed in parallel using an antibody raised against the target protein or an antibody raised against the tag. Fifth, the protein can be affinity-purified with the antibody and analyzed by mass spectrometry. Uhlen and colleagues recommend using at least one of these pillars as a minimal criterion for antibody specificity. However, our own experience taught us that the parallel employment of several strategies (overexpression, use of recombinant proteins and knockout) is necessary for confident proof of antibody specificity. A useful resource to select antibodies is the antibodypedia, a repository of already validated antibodies which reports on primary data, publications and commentaries on commercially available antibodies (https://www.antibodypedia.com/).

Our observation that MRT67307 inhibits CYLD phosphorylation in IKKε and TBK1 deficient cells indicates a role for other MRT67307-sensitive kinases in TCR signaling. While MRT67307 was first described as an inhibitor of TBK1 and IKKε (IC50 value of 19 and 160 nM, respectively) (Clark et al., 2011a), several AMP-activated protein kinase (AMPK)-related kinases were later described as additional targets: MAP/microtubule affinity-regulating kinases (MARK1)-4 (IC50 value of 27–52 nM), NUAK family kinase 1 (NUAK1) (IC50 value of 230 nM), and the salt inducible kinases (SIK) (IC50 value of 250, 67, and 430 nM for SIK1, SIK2, and SIK3, respectively) (Clark et al., 2011a, 2012). Moreover, MRT67307 is also an inhibitor of Unc-51 Like Autophagy Activating Kinase (ULK) 1 and ULK2 with IC50 values of 45 and 38 nM, respectively (Petherick et al., 2015). In addition, the MRC PPU International Centre for Kinase Profiling reported *in vitro* specificity screens for several inhibitors (http://www.kinase-screen.mrc.ac.uk/kinase-inhibitors), showing that MRT67307 not only inhibits IKKε/TBK1 and the AMPK-related kinases MARK, MELK and NUAK, but also Mixed Lineage Kinase (MLK) 1 and MLK3, Janus kinase (JAK) 2, and Ca(2+)/calmodulin-dependent protein kinase kinase β (CamKKβ). It will be interesting to further investigate if any of the above mentioned kinases is responsible for the inducible phosphoprotein signal detected by the phospho(Ser418)-CYLD-specific antibody. Moreover, the non-specificity of MRT67307 also implies that numerous studies using this compound as an IKKε/TBK1 inhibitor may have to be revisited (summarized in Table 5). The above findings illustrate the importance of validating small compound kinase inhibitors for specificity. Much valuable information is publicly available from selectivity profile data of commonly used kinase inhibitors screened against panels of kinases (Davies et al., 2000; Bain et al., 2003, 2007; Fabian et al., 2005; Fedorov et al., 2007; Bamborough et al., 2008; Karaman et al., 2008). Additional information on kinase screens or kinome scans can be found on http://www.kinase-screen.mrc.ac.uk/kinase-inhibitors or http://lincs.hms.harvard.edu/kinomescan/, respectively. Also specific recommendations on the most optimal inhibitor or combinations of inhibitors to study a number of selected kinases have been published (Davies et al., 2000; Bain et al., 2003, 2007). For example, using at least two structurally different inhibitors or cells expressing a drug resistant mutant of the kinase to validate findings is much desired. We would also recommend using genetic knockout or siRNA mediated knockdown of the specific kinase to complement data obtained from inhibitor studies.

**Table 5:** Publications in which MRT67307 was used.

**References**	**Intended target**	**Other targets**	**IC50 [nM]**	**Effect of inhibitor treatment**	**Confirmation of inhibitor effects**	**Concentration**
Clark et al., 2011a	TBK1/IKKε		19/160	Inhibition of PolyI:C induced IRF3 phosphorylation, but not JNK and p38 phosphorylation and reduced LPS-induced IFNβ production in macrophages; Enhanced NF-κB activation in response to IL-1 or TNF and PolyI:C or LPS in MEF cells and macrophages, respectively		2μM
Gleason et al., 2011	TBK1/IKKε			Reduced OPTN phosphorylation in LPS-treated BMDMs in combination with TAK1 inhibitor 5Z-7-oxozeanol.		
Smith et al., 2011	TBK1/IKKε			Inhibition of LPS-induced Pellino-1 induction and IRF3 phosphorylation in BMDMs; Reduced IKKε and TBK1 dependent Pellino 1 phosphorylation *in vitro*; Reduced LPS- or PolyI:C-induced Pellino 1 activation in macrophages		2μM
Clark et al., 2011b	TBK1/IKKε			Increased TBK1 and IKKε activationin Pam3CSK4 stimulated BMDMs	IKKε and TBK1 knockout MEF cells	2μM
Dzamko et al., 2012	TBK1/IKKε			Inhibition of phosphorylation of LRRK2 in Pam3CSK4 stimulated RAW 264.7 cells		2μM
Liu et al., 2012	TBK1			Abrogation of thapsigargin-dependent IRF3 phosphorylation in MEF cells		2μM
Bruni et al., 2013	TBK1/IKKε			Reduced ERK phosphorylation in response to PolyI:C in BMDMs	IKKε and TBK1 knockout BMDMs	2μM
Jiang et al., 2015	TBK1/IKKε			Decreased IFN-λ1 mRNA expression in short dsRNA-stimulated human monocyte-derived DCs		0.1–10μM
Zhu et al., 2014	TBK1/IKKε			Impaired CYLD (Ser418) phosphorylation upon co-expression with IKKε/TBK1 in HEK cells; decreased LPS-induced expression of IFNβ1, CCL5 and IL-6 in RAW 264.7 cells; impaired viability of KRAS-dependent A549 and HCC44 cells	genetic evidence (Tbk1^−/−^ MEFs), independent inhibitor (CYT387)	0.1–10μM
Lopez-Pelaez et al., 2014	TBK1/IKKε			Inhibition of *in vitro* phosphorylation of IRF5 by TBK1; no effect on nuclear translocation of IRF5 upon CL097 (TLR7/8) in RAW 264.7 cells		2μM
Awuh et al., 2015	TBK1			Decreased NF-κB and IRF-1 nuclear translocation in response to *M. avium* infection in monocyte derived macrophages; Increased *M. avium* growth	independent inhibitor (BX795)	5μM
Pillai et al., 2015	TBK1			Inhibition of mitotic progression and formation of multinucleate cells in H460 and HeLa cells	independent inhibitor (BX795) (knockdown in additional experiments)	2μM
Yu et al., 2015	TBK1			Inhibition of AKT degradation in anti-CD3/CD28 stimulated CD4^+^ T cells in the presence of cycloheximide		2μM
Bruni et al., 2015	TBK1/IKKε			Increased viral RNA production in pDCs infected with yellow fever life vaccine and reduced type I IFN production		1μM
Saric et al., 2016	TBK1			No effect on LPS-induced lysosome tubulation in RAW 264.7 macrophages; inhibition of LPS-induced IRF3 phosphorylation		2μM
Heo et al., 2015	TBK1			Inhibition of recruitment of OPTN, NDP52 and SQSTM1 to depolarized mitochondria in Antimycin A and Oligomycin A treated HeLa cells	TBK1^−/−^ cells	2μM
Swamy et al., 2015	TBK1/IKKε			Reduced *Ifnα* and *Ifnl* mRNA induction, but no effect on *Ifnγ* upregulation in CD3-stimulated intestinal intraepithelial lymphocytes		1μM
Schweitzer et al., 2016	TBK1/IKKε			Marginal decrease in phosphorylation of IκBα and RelA (Ser468) in the cytosol and RelA nuclear translocation in TNF-stimulated HeLa cells		10μM
Achard et al., 2017	TBK1/IKKε			Impaired IFNα production and TRAIL expression in response to measles virus infection or R837 (TLR7 agonist) stimulation in plasmacytoid DCs; Reduced TRAIL expression in response to type I IFN		8μM
Clark et al., 2012	SIK1		250	Increased TLR-induced expression of anti-inflammatory cytokines (IL-10 and IL-1ra) in macrophages	Structurally different inhibitors (MRT199665, HG-9-91-01, KIN112), genetic evidence (LKB1^−/−^ macrophages)	2μM
	SIK2		67			
	SIK3		430			
		MARK1-4	27-52			
		NUAK1	230			
MacKenzie et al., 2013	SIK			Increased LPS-induced IL-10 mRNA in BMDMs	Independent inhibitor (KIN112)	2μM
Petherick et al., 2015	ULK1		45	Reduced amino acid starvation-induced ATG13 phosphorylation and autophagy in MEF cells	Structurally different inhibitor (MRT68921)	10μM
	ULK2		38			
Lazarus and Shokat, 2015	ULK1/2			Inhibition of autophagy (LC3 II accumulation) in HeLa cells	Independent inhibitor (BX795)	4μM
Draz et al., 2017	ULK1/2			Sensitization of LNCaP and C42B cells to cell death in the presence of subtoxic concentrations of 3,3′-diindolylmethane		10μM

In conclusion, the present case study clearly illustrates several pitfalls when using antibodies and small compound inhibitors. There is a clear need for better validation of antibodies and small compound inhibitors to make results more reliable and reproducible. More specifically, some previously published data making use of MRT67307 or phospho(Ser418)-CYLD specific antibodies may have led to wrong conclusions or at least need to be validated by independent approaches if not yet done so. Finally, our results also reveal that TCR stimulation results in the IKKε/TBK1-independent phosphorylation of an unknown 110 kDa protein, which can be inhibited by MRT67307, and which masks the detection of phosphorylated CYLD upon western blotting with a phospho(Ser418)-CYLD-specific antibody. The identification of this phosphorylated protein as well as the responsible kinase that is inhibited by MRT67307 will be the topic of future studies.

## Author contributions

ML, JS, and RB designed the research. ML and MK performed the experiments. ML analyzed the data. ML, JS, and RB wrote the paper.

### Conflict of interest statement

The authors declare that the research was conducted in the absence of any commercial or financial relationships that could be construed as a potential conflict of interest. The reviewer NR and handling Editor declared their shared affiliation.

## Supplementary material

Figure S1 CYLD does not show a band shift indicative of phosphorylation upon stimulation with PMA/I or TNF. Different Jurkat T cell clones were stimulated with 200 ng/ml PMA and 1μM Ionomycin or 1,000 U/ml TNF for the indicated times. Protein levels were determined by immunoblotting. Jurkat-E (from Shigekazu Nagata, Osaka), Jurkat E6.1 (ATCC), Jurkat TNFR2 (gift from Nicholas Harper, Leicester).
