# Supplementary figures and images for: Importance of Validating Antibodies and Small Compound Inhibitors Using Genetic Knockout Studies—T Cell Receptor-Induced CYLD Phosphorylation by IKKε/TBK1 as a Case Study
Source: Front Cell Dev Biol. 2018 Apr 10;6:40. doi: 10.3389/fcell.2018.00040 (PMC5932415; doi:10.3389/fcell.2018.00040)

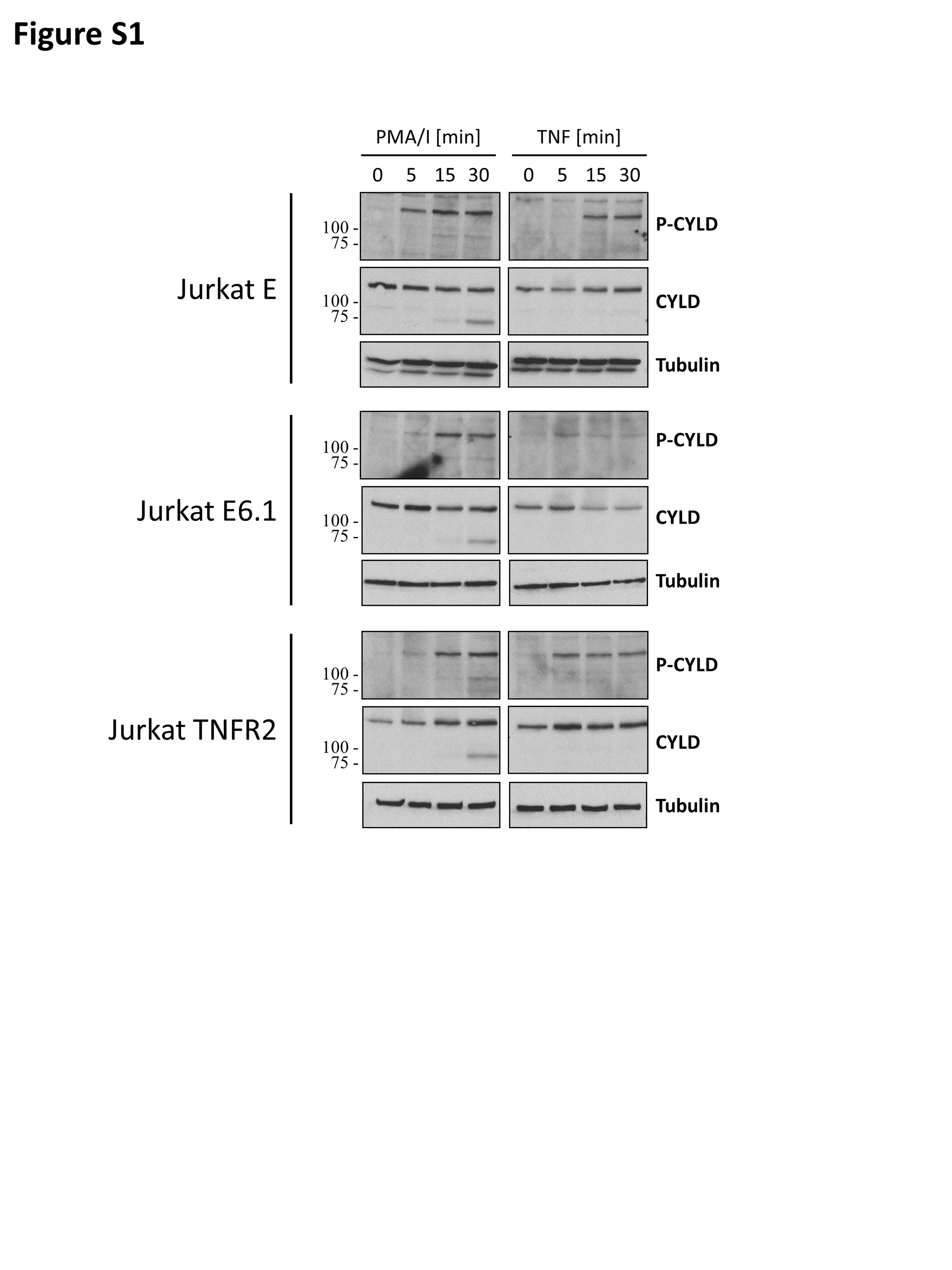

Supplement: Figure S1 — CYLD does not show a band shift indicative of phosphorylation upon stimulation with PMA/I or TNF. Different Jurkat T cell clones were stimulated with 200 ng/ml PMA and 1μM Ionomycin or 1,000 U/ml TNF for the indicated times. Protein levels were determined by immunoblotting. Jurkat-E (from Shigekazu Nagata, Osaka), Jurkat E6.1 (ATCC), Jurkat TNFR2 (gift from Nicholas Harper, Leicester). [file Image1.TIF]
